# The validity and reliability of remote diabetic foot ulcer assessment using mobile phone images

**DOI:** 10.1038/s41598-017-09828-4

**Published:** 2017-08-25

**Authors:** Jaap J. van Netten, Damien Clark, Peter A. Lazzarini, Monika Janda, Lloyd F. Reed

**Affiliations:** 1 School of Clinical Sciences, Institute for Health and Biomedical Innovation, Queensland University of Technology, Brisbane, QLD Australia; 2 Wound Management Innovation Cooperative Research Centre, West End, QLD 4101 Australia; 3 Department of Podiatry, Metro North Hospital & Health Service, Queensland Health, Brisbane, QLD Australia; 4 Allied Health Research Collaborative, Metro North Hospital & Health Service, Queensland Health, Brisbane, QLD Australia; 5 School of Public Health and Social Work, Institute for Health and Biomedical Innovation, Queensland University of Technology, Brisbane, QLD Australia

## Abstract

Despite their potential for telemedicine in diabetic foot ulcer treatment, diagnostic accuracy of assessment of diabetic foot ulcers using mobile phone images is unknown. Our aim was to determine the validity and reliability of remote diabetic foot ulcer assessment using mobile phone images. Fifty diabetic foot ulcers were assessed live and photographed. Five independent observers remotely assessed the mobile phone images twice for presence of nine clinical characteristics and three treatment decisions. Positive likelihood (LLR+) and negative likelihood (LLR−) ratios were calculated for validity. Multirater Randolph’s and bi-rater Bennet kappa values were calculated for reliability. LLR+ ranged from 1.3–4.2; LLR− ranged from 0.13–0.88; the treatment decision ‘peri-wound debridement’ was the only item with ‘strong diagnostic evidence’. Inter-observer reliability kappa ranged from 0.09–0.71; test-retest reliability from 0.45–0.86; the treatment decision ‘peri-wound debridement’ was the only item with ‘adequate agreement’. In conclusion, mobile phone images had low validity and reliability for remote assessment of diabetic foot ulcers and should not be used as a stand-alone diagnostic instrument. Clinicians who use mobile phone images in clinical practice should obtain as much additional information as possible when making treatment decisions based on these images, and be cautious of the low diagnostic accuracy.

## Introduction

Diabetic foot ulcers are a major health problem with significant morbidity and mortality^1,2^. Recent global pooled estimates indicate 3.4% of all inpatients have a diabetic foot ulcer, and 1.5% a diabetes-related amputation procedure at any given time^3^. These diabetic foot ulcers lead to major healthcare expenditures and a reduction in people’s quality of life^4–6^, and nearly all amputations in people with diabetes are precipitated by a non-healing foot ulcer^1,2^.

People with diabetic foot ulcers require frequent evidence-based treatment in highly-skilled interdisciplinary foot clinics^2,7^. This typically involves weekly ulcer treatment visits to the foot clinic and additional self-care of the ulcer at home between clinic visits by themselves, their carers or home care nurses^2,7^. As treatment to achieve ulcer healing often lasts for three months or more^8^, this requires multiple clinic visits. These frequent clinic visits can often be a burden to patients in terms of their time, effort and finances, especially for people living in rural and remote areas or people with travel difficulties. Additionally, weekly visits may still not be enough to detect deterioration of foot ulcers in sufficient time to prevent hospitalisation or amputation, as infections may develop and progress to life-threatening severe infections over just days^9,10^.

To overcome these limitations and to empower people with diabetic foot ulcers in their self-care away from the clinic, various telemedicine systems have been investigated. The cornerstone of these telemedicine systems is clinical assessment of digital photographic images. Three studies used wound assessment platforms with uploaded high-resolution images from digital cameras^11–13^. However, these platforms can only be used by health professionals, thereby not increasing patient empowerment. Other studies investigated specially developed advanced stand-alone imaging devices^14–17^. These have shown high reliability for measuring ulcer area size and high validity for diagnosing the presence of an ulcer or callus. However, measuring these characteristics is not enough to reduce clinic visits or improve timely detection of limb-threatening disease. For that, more detailed clinical characteristics or treatment decisions (such as the diagnosis of infection, presence of exudate, the need for debridement) need to be assessed. These have only been investigated scarcely, with low validity found^15,18^.

In addition to these validity and reliability findings, all telemedicine systems investigated were expensive and require highly technical equipment^14–17^. Anecdotally, clinicians and patients have overcome these practical disadvantages by using mobile phones to capture photographic images of ulcers instead of using these wound assessment platforms. We have seen mobile phone images used for this purpose routinely in multiple daily clinical practice situations; for example by home-care nurses or by patients for unofficial ‘telemedicine’ consultations with an interdisciplinary foot clinic.

To our knowledge, only two studies have investigated the use of mobile phone images for diabetic foot ulcer assessment^19,20^. One of them investigated ulcer area measurement only, but did not investigate assessment of any other clinical characteristics^19^. The other study, by Rasmussen and colleagues, compared a standard live clinical assessment with a remote assessment based on images from both an i-phone and a new imaging device^20^. They reported low kappa values for the diagnosis of sixteen clinical characteristics, based on the combined assessment of two remote observers. However, kappa values are a measure of agreement reflecting reliability and are not representative of validity; whereas likelihood ratios, sensitivity, and specificity are the statistics that should be used to analyse validity^21,22^, but these were not reported in this paper^20^. Perhaps even more importantly, any treatment decisions based on the clinical characteristics assessed in the i-phone images were also not assessed. Limited agreement in assessment of clinical characteristics may in fact still lead to similar treatment decisions; however this is unknown. Therefore, despite their potential for use as a practical telemedicine system in diabetic foot ulcer treatment, diagnostic validity and reliability of assessment of diabetic foot ulcers using mobile phone images is unknown.

The aim of this study was to determine the validity and reliability of remote assessment of diabetic foot ulcer clinical characteristics and treatment decisions using photographs produced on a mobile phone in comparison to a reference standard live clinical assessment.

## Methods

### Design

A prospective diagnostic validity and reliability study design was used. The Standards for Reporting Diagnostic accuracy studies (STARD) were used for reporting the study^23^.

### Participants

Eligible participants were adults with an existing diabetic foot ulcer who provided written informed consent. People with a cognitive deficit that would impair their ability to read and write or complete certain technical aspects of the study were excluded. Participants were recruited between July and September 2015 from four Diabetic Foot Clinics within the Metro North Hospital and Health Service and the Metro South Hospital and Health Service, Brisbane, Queensland, Australia.

Eligible remote observers were five registered clinical podiatrists with different levels of experience in the management of diabetic foot ulcers who provided written informed consent. For the purposes of this study, observer experience levels in diabetic foot ulcer management were differentiated using formal Health Practitioner Level (HPL) of employment appointment^24^ and years of experience specifically treating people with diabetic foot ulcers. The five remote observers recruited were: observer 1, a ‘specialist clinician (HPL5)’ podiatrist with 13 years of diabetic foot experience; observer 2, a ‘senior clinician (HPL4)’ podiatrist with 10 years of diabetic foot experience; observer 3, a HPL4 podiatrist with 8 years of diabetic foot experience; observer 4, a HPL4 podiatrist with 6 years of diabetic foot experience; and observer 5, a newly graduated ‘clinician (HPL3)’ podiatrist with no diabetic foot experience. The observers were recruited from Diabetic Foot Clinics that were independent of the four Diabetic Foot Clinics that the participants were recruited from, and therefore, none of them were involved in the clinical care of the participants.

The reference standard live clinical assessment was the criterion measure used for the purposes of this study and defined as an in-person clinical assessment by a registered clinical podiatrist with significant experience in the management of diabetic foot ulcers. A full-time Podiatry Clinical Educator in the management of diabetic foot ulcers was chosen to perform the reference standard live clinical assessments. The educator was responsible for diabetic foot ulcer education and training at the Queensland University of Technology and had been responsible for new graduate clinical support within Queensland Health in the management of diabetic foot ulcers for the past five years. Prior to this appointment, the clinical educator had been a HPL5 specialist clinical podiatrist with four years of specific diabetic foot experience.

Based on the ability to find kappa values of >0.40^25^, with an anticipated 30–50% prevalence of the clinical characteristics, 80% power and alpha <0.05, a sample size of 50 participants was needed^26^, which is somewhat larger compared to the samples sizes ranging from 20–36 participants in related studies^14–16^.

### Procedures

After providing informed consent, participants underwent the reference standard live clinical assessment. This visual assessment comprised of completing the 12 items from the study form (Table 1). The items from the study form were similar to those used in the study by Bowling and colleagues^14^, and included 9 clinical characteristic and 2 treatment decision items. One additional treatment decision item was added: “If this person wasn’t seen in the clinic, select the time-frame for when this person should be seen in-person”. This question was used to represent the standard final treatment decision of any consultation on the urgency of follow-up treatment. In this case when a clinician or patient sends mobile phone images of a diabetic foot ulcer to an expert clinician seeking urgent clinical decision advice on when they should next attend the clinic for care: the answers “same day” and “next day” were categorised as “urgent treatment by a health professional required”, with the remaining answers categorised as “no urgent treatment by a health professional required”.

**Table 1:** Study form with clinical questions for live and remote assessment.

Clinical characteristic assessment	Answer
Is there evidence of some granulation tissue?	Yes/No
Is there evidence of ischemia?	Yes/No
Is there evidence of wound infection?	Yes/No
Does the wound bed contain slough?	Yes/No
Can you see tendon or bone in the wound bed?	Yes/No
Does the wound appear to be tracking or tunneling?	Yes/No
Is there evidence that the wound is moist or exuding?	Yes/No
Is there presence of wet or dry gangrene?	Yes/No
Is there evidence of surrounding cellulitis or erythema?	Yes/No
**Treatment decisions**	
Is there evidence that debridement of the wound would improve healing?	Yes/No
Is there evidence that debridement of the skin around the wound would improve healing?	Yes/No
If this person wasn’t seen in the clinic, select the time-frame for when this person should be seen in-person^a^	Same day/Next day/Within 3 days/Within 1 week/Within 2 weeks/>2 weeks^a^

Note: ^a^This question was used to define urgency of treatment, with “same day” and “next day” defined as “urgent treatment by a health professional required”, and the remaining answers as “no urgent treatment by a health professional required”.

Additionally, pre-existing clinical information of the Queensland High Risk Foot Form (QHRFF) was used^25^. The QHRFF is a reliable and valid research tool for foot disease with substantial and near-perfect inter-observer agreement, has been extensively described elsewhere, and is standard of care in Queensland^25^. For the purpose of the current study, the age, gender, diabetes, co-morbidities, foot disease history (all self-reported by patients, as described in the QHRFF), most recent HbA1c, and clinical diagnoses of peripheral neuropathy (“present” or “absent”) and peripheral artery disease (“nil”, “moderate”, or “critical”) were used^25^.

**Figure 1:**
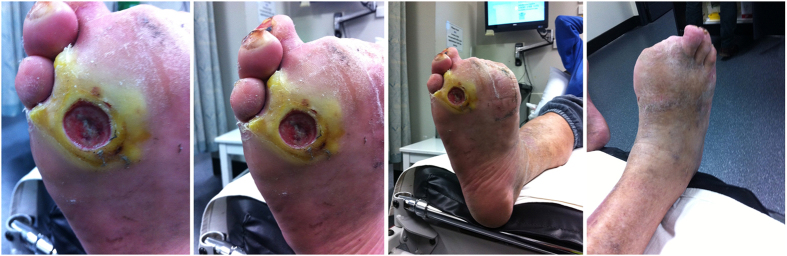
Example of the four mobile phone images taken for remote assessment.

Immediately following the live clinical assessment, four non-identifiable photos of the ulcer were taken by an independent research assistant using an i-phone 4 (Apple Inc, Cupertino, CA, United States of America), with image resolution 1936 × 2592 pixels. When more than one ulcer was present, the largest ulcer was selected as the target ulcer and placed in the centre of the field of view. Research assistants were provided with 1 hour training in the use of the mobile phone for taking diabetic foot ulcer images before the study, using a standard presentation and a foot model with a foot ulcer to practice. They had limited or no clinical experience in working with people with diabetic foot ulcers, to mirror the similar experience of photo taking by patients, relatives or home-care nurses without specific diabetic foot ulcer experience, and thereby to avoid potential bias from having an experienced clinician taking the photos. The four photos included: i) close-up of the ulcer ensuring that the majority of the wound is in the frame; ii) a mid-way shot, positioning the camera to capture at least a 4–6 cm border around the wound to assess status of skin and tissue integrity; iii) a distant shot showing the foot in its entirety (with the wound in view); and iv) a mid-way shot, positioning the camera to capture the opposite side of the foot from where the wound is situated to identify any significant infection or tissue quality and/or colour changes. See Fig. 1 for an example. The mobile phones were not connected to a telecommunications network and were only used for the purposes of taking images of ulcers for this study.

After all measurements had been taken, the remote observers were provided with the mobile phone images and the additional clinical information from the QHRFF. The information from the QHRFF was provided as it was thought that remote mobile phone assessment will not occur in isolation in daily clinical practice, but with patients from whom it can be expected that some baseline information would be available to the clinician. The observers were asked to complete the 12-item study form for the target ulcer (Table 1) and were allowed to manipulate the touch screen to further expand the images to more accurately reflect current practice with mobile phone image use. All observers were blinded from any of the other observers’ remote assessments. Additionally, the observers were asked if the image quality allowed them to adequately assess the target ulcer, with their options ranging from 1 (strongly agree) to 5 (strongly disagree). A minimum of two weeks after their first assessment, the observers completed the same assessment of the same mobile phone images again, without having access to their previous assessment.

The procedures were approved by Human Research Ethics Committee of the Prince Charles Hospital, Brisbane, Queensland, Australia (HREC/14/QPCH/204). All procedures were in accordance with the principles of the Declaration of Helsinki.

### Outcome Measures

Validity of remote mobile phone assessment of diabetic foot ulcers was analysed by calculating the following diagnostic values: sensitivity, specificity, positive likelihood ratio (LLR+) and negative likelihood ratio (LLR−) between the reference standard live clinical assessment and the first assessment made by each of the individual remote observers. Although live clinical assessment can be inherently subjective, it is the internationally accepted reference standard for clinical characteristics, clinical decision-making and treatment planning^27^, has been demonstrated to be reliable using the QHRFF^24^ and has been used in similar studies^14,15,18,20^. It is also the most reflective reference standard of daily practice in which remote mobile phone assessment may be used.

The primary endpoints chosen were LLR+ and LLR−, as they provide the most meaningful outcome for clinical decision-making^21,22^. An LLR+ >5 or LLR− <0.2 indicated “strong” diagnostic evidence, and an LLR+ >10 or LLR− <0.1 indicated “convincing” diagnostic evidence^21,22^. Sensitivity and specificity were secondary endpoints. These values need to be “high” to either rule out or confirm a disease, but as these values also depend on prevalence no generally agreed on hard cut-off score is available^21,22^. We chose >80% for sensitivity and specificity as “high”^21^.

Reliability of remote mobile phone assessment of diabetic foot ulcers was analysed by calculating inter-observer and test-retest reliability. Inter-observer reliability was determined by calculating free marginal multirater Randolph’s kappa values^28,29^, and test-retest reliability was determined by calculating free marginal bi-rater Bennett kappa values^29^. Free marginal kappa values were calculated because raters’ distributions of cases into categories were not restricted for any of the observations made. Values > 0.7 were considered “adequate” agreement^28,30^. Prevalence of the clinical characteristics assessed during live assessment was unknown to the observers, and the assumption was made that it could also not be reliably guessed based on clinical experience.

### Data analysis

SPSS version 23.0 software (IBM Corporation, Armonk, NY, USA) was used for analysis of descriptive characteristics. For validity, the sensitivity, specificity (both including 95% confidence intervals), LLR+ and LLR− of live vs. remote assessment were calculated per remote observer using Review Manager (RevMan) Version 5.3 (The Nordic Cochrane Centre, The Cochrane Collaboration, Copenhagen, Denmark) and Microsoft Excel (Microsoft Corporation, Redmond, WA, USA). Mean values over the five observers were calculated and presented. There were no missing values. For inter-rater and test-retest reliability, free marginal kappa values were calculated using the online kappa calculator at http://justusrandolph.net/kappa/. One observer missed a second assessment for two clinical characteristics (‘infection’ in one patient, ‘slough’ in another patient). Test-retest reliability was calculated based on the observations for the remaining 49 participants for those two clinical characteristics in that observer. There were no further missing values.

### Data availability

The datasets generated during and/or analysed during the current study are available from the corresponding author on reasonable request.

## Results

### Participants

A total of 53 consecutive people with diabetes mellitus and a foot ulcer provided informed consent for participation in the study. The ulcers of three persons had healed in the short time-frame between providing informed consent and the study visit and these persons were excluded, leaving a total of 50 participants. Table 2 displays the participant characteristics including a mean (standard deviation) age of 61 (11), 80% were male, diabetes duration of 20 (13) years, and 60% of the ulcers was located on the plantar side of the foot.

**Table 2:** Participant characteristics (n = 50).

Personal and medical characteristics	
Gender (male – female)	80% (n = 40)–20% (n = 10)
Age (years)	61 (11)
Diabetes type (type 1–type 2)	22% (n = 11)–78% (n = 39)
Years with diabetes^a^	20 (12.6)
HbA1c (mmol/L)^b^	8.1 (1.6)
Peripheral neuropathy	100% (n = 50)
Peripheral artery disease (nil – moderate – severe)	48% (n = 24)–42% (n = 21)–10% (n = 5)
**Medical history**	
Previous foot ulcer	96% (n = 48)
Previous foot amputation	62% (n = 31)
Foot deformity	96% (n = 48)
Hypertension	70% (n = 35)
Dyslipidaemia	38% (n = 19)
Cardiovascular disease	38% (n = 19)
Chronic kidney disease	30% (n = 15)
End-stage renal failure	2% (n = 1)
**Ulcer characteristics**	
Type (neuropathic – neuroischemic)	48% (n = 24)–52% (n = 26)
Location (left – right)	54% (n = 27)–46% (n = 23)
Location (plantar – dorsal – other)	60% (n = 30)–10% (n = 5)–30% (n = 15)
Location (toes – forefoot – midfoot–heel)	38% (n = 19)–32% (n = 16)–20% (n = 10)–10% (n = 5)
**Clinical characteristics** ^**c**^	
Granulation	66% (n = 33)
Ischemia	22% (n = 11)
Infection	18% (n = 9)
Slough	42% (n = 21)
Tendon or bone	0% (n = 0)
Tracking or tunnelling	28% (n = 14)
Moist or exuding	44% (n = 22)
Wet or dry gangrene	0% (n = 0)
Cellulitis or erythema	22% (n = 11)
**Treatment decisions** ^**c**^	
Wound debridement	70% (n = 35)
Peri-wound debridement	70% (n = 35)
Urgent treatment	44% (n = 22)

Note: Values are % (n) or Mean (Standard Deviation); ^a^Years with diabetes was missing for five participants; ^b^HbA1c was missing for 23 participants. ^c^See Table 1 for formulation of the questions, all answers were ‘yes’ or ‘no’ with percentage ‘yes’ given in this table.

### Prevalence during live assessment

Prevalence of seven of the nine clinical characteristics ranged from 18% to 66% during live assessment (Table 2). No participant had the two remaining clinical characteristics (“tendon or bone visible” or “wet or dry gangrene”) during live assessment. These two clinical characteristics were excluded from further analyses, as diagnostic values cannot be calculated when the denominator is zero.

Prevalence of the requirement for the treatment decisions of wound and peri-wound debridement were both 70% during live assessment (Table 2). The prevalence for urgent treatment was determined for 44% during live assessment (Table 2).

### Prevalence during remote assessment

During remote assessment, prevalence of the seven clinical characteristics ranged from 6% to 80% (see Supplementary Table 1). Prevalence of the requirement for the treatment decisions of wound debridement ranged from 62% to 98%, and for the treatment decision of peri-wound debridement from 78% to 100% over the five remote observers (see Supplementary Table 1). The prevalence for urgent treatment ranged from 0% to 66% over the five remote observers (see Supplementary Table 1).

### Validity

**Figure 2:**
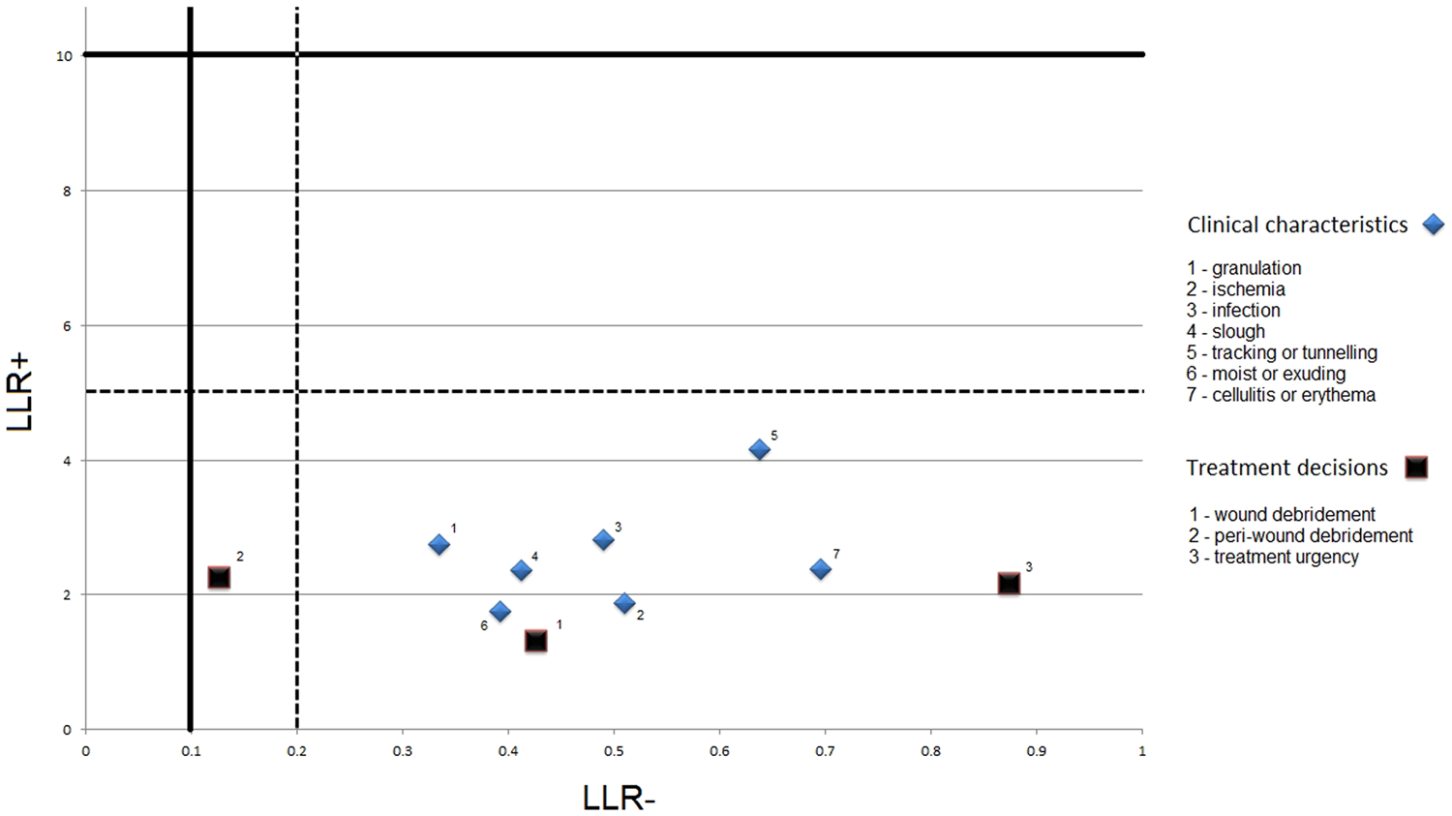
Positive and negative likelihood ratios of remote assessment of diabetic foot ulcers. Legend: Positive (LLR+) and Negative (LLR−) Likelihood ratios of remote assessment of diabetic foot ulcers, when compared to ‘reference standard’ live clinical assessment for 7 clinical characteristics and 3 treatment decisions. Values left from or above the solid line indicate ‘convincing diagnostic evidence’, values left or above the dotted line indicate ‘strong diagnostic evidence’^21,22^. See Table 1 for the formulation of the questions to assess clinical characteristics and treatment decisions.

LLR+ values ranged between 1.3 and 4.2, with no items having “strong” or “convincing” diagnostic evidence for LLR+ (Fig. 2). LLR− values ranged between 0.13 and 0.88 (Fig. 2), with one item having “strong” diagnostic evidence for LLR− (treatment decision of peri-wound debridement; LLR−: 0.13). The remaining LLR− values ranged between 0.33 and 0.88 (Fig. 2), not resulting in strong diagnostic evidence^21,22^.

**Figure 3:**
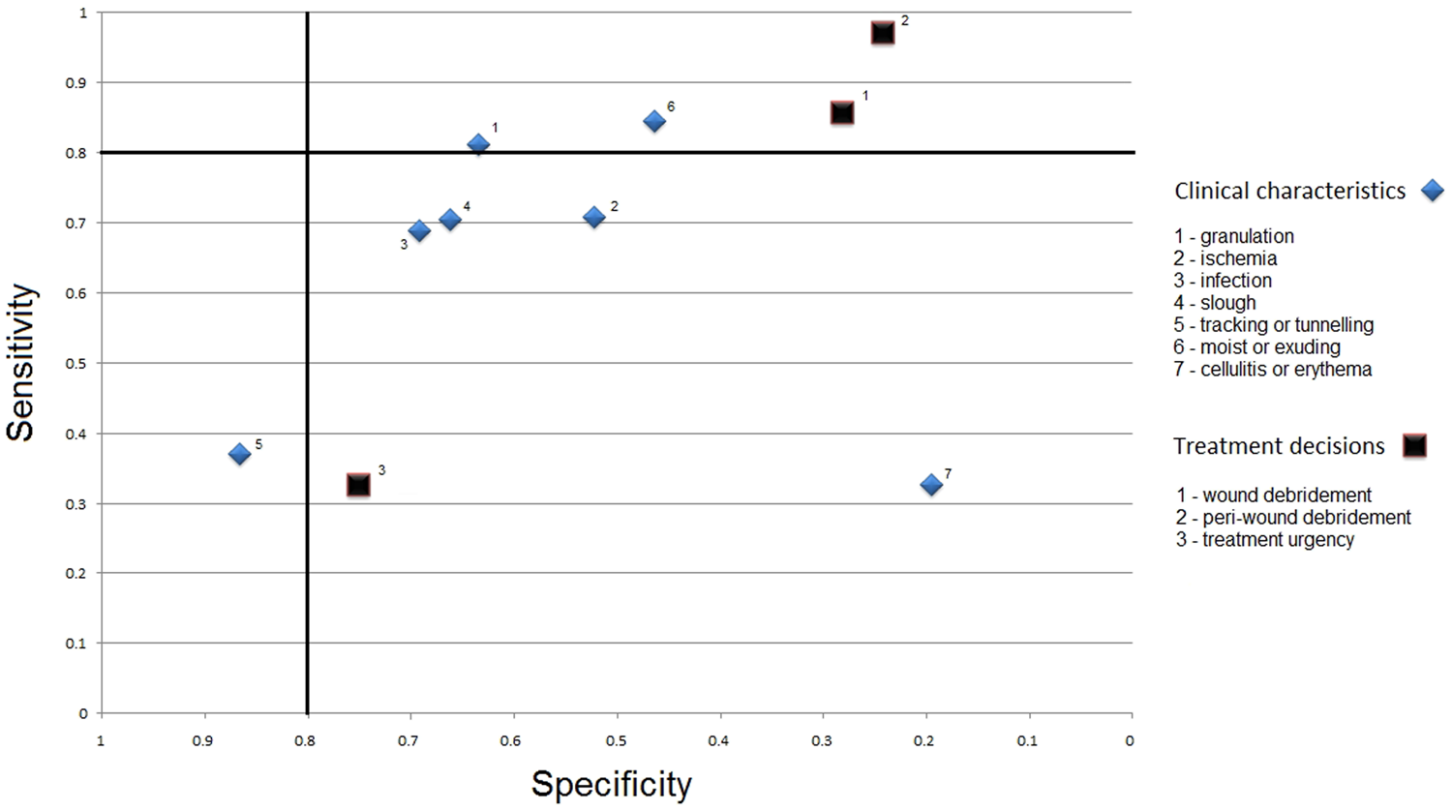
Sensitivity and specificity of remote assessment of diabetic foot ulcers. Legend: Sensitivity and specificity of remote assessment of diabetic foot ulcers, when compared to ‘reference standard’ live clinical assessment for 7 clinical characteristics and 3 treatment decisions. Values left from or above the solid line indicate ‘high diagnostic evidence’^21^. See Table 1 for the formulation of the questions to assess clinical characteristics and treatment decisions.

Sensitivity findings ranged from 32% to 97% (Fig. 3), with four items having “high” sensitivity (two clinical characteristics: “granulation tissue” and “moist or exuding wound”; two treatment decisions: “wound debridement” and “peri-wound debridement”). Specificity findings ranged from 20% to 87% (Fig. 3), with one item having “high” specificity (clinical characteristic: “tracking or tunnelling wound”). None of the items recorded both “high” sensitivity and specificity.

All absolute agreement, sensitivity and specificity values (including 95% confidence intervals) per observer are given in Supplementary Table 2 for further information. Mean values per observer did not show major differences between observers (see Supplementary Table 2). Also, the most experienced observer scored similar to the least experienced observer (see Supplementary Table 2).

### Reliability

Inter-observer reliability kappa values ranged from 0.09 to 0.71, with only the treatment decision item of peri-wound debridement reaching “adequate agreement” (Table 3). Mean test-retest reliability kappa values of the five observers ranged from 0.45 to 0.86, with again peri-wound debridement the only item reaching “adequate agreement” (Table 3).

**Table 3:** Reliability of remote mobile phone diabetic foot ulcer assessment.

**Clinical characteristics** ^a^	Inter-observer reliability		Test-retest reliability	
	S	%	S	%
Granulation	0.29	64	0.62	81
Ischemia	0.09	54	0.47	74
Infection	0.16	58	0.47	73
Slough	0.27	64	0.45	73
Tracking or tunnelling	0.49	74	0.64	82
Moist or exuding	0.37	69	0.50	75
Cellulitis or erythema	0.37	68	0.54	77
**Treatment decisions** ^a^				
Wound debridement	0.43	72	0.69	84
Peri-wound debridement	0.71	86	0.86	93
Urgent treatment	0.22	60	0.48	74

Note: S = free marginal Randolph’s kappa coefficient; % = percentage total agreement. For test-retest reliability mean kappa over the five observers is presented, for individual scores see Fig. 4. ^a^See Table 1 for formulation of the questions.

**Figure 4:**
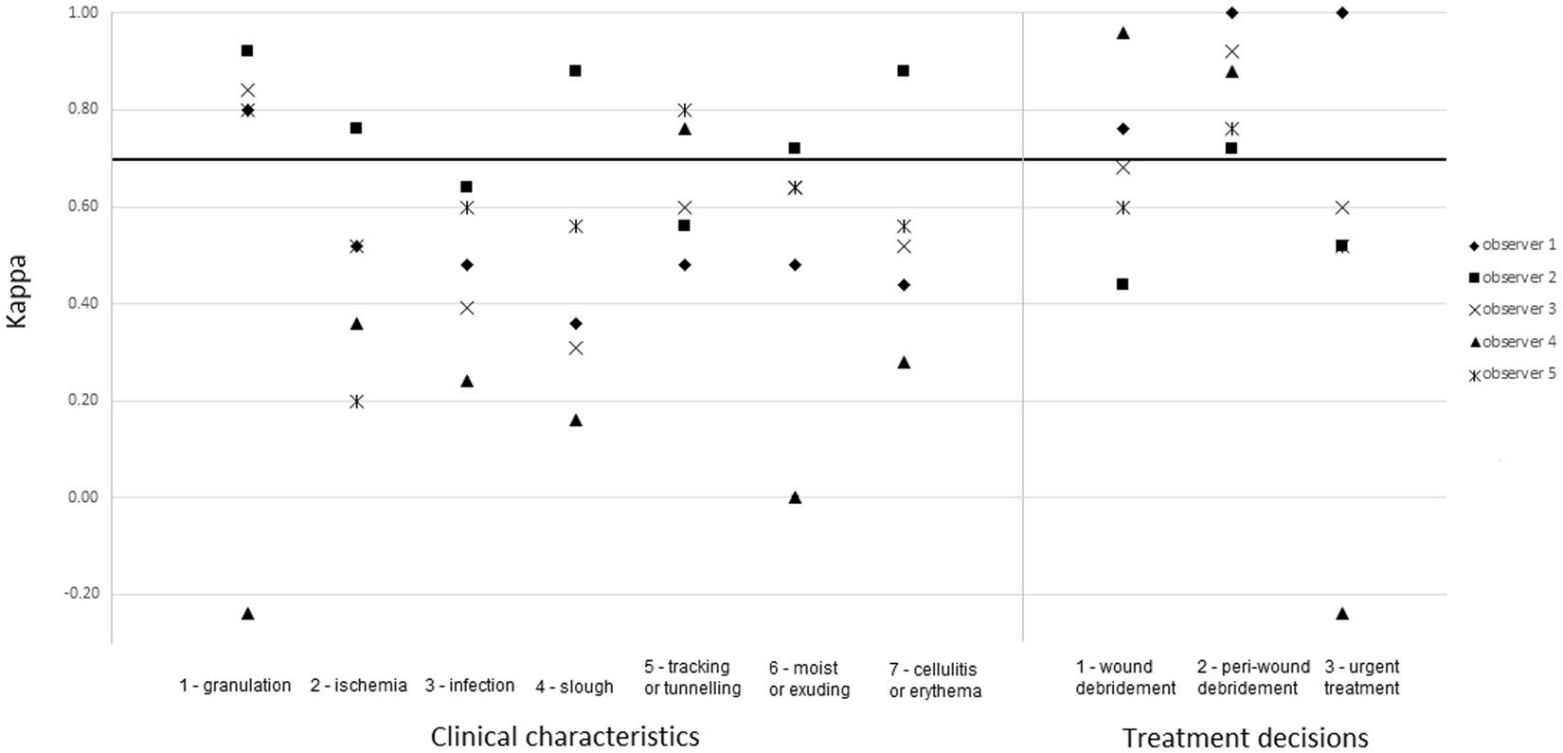
Test-retest reliability of remote assessment of diabetic foot ulcers per observer. Legend: Scores above the solid black (0.7) indicate ‘adequate agreement’^21,22^. See Table 1 for the formulation of the questions to assess clinical characteristics and treatment decisions.

Individual test-retest reliability scores per observer are presented in Fig. 4. All observers scored >0.7 for peri-wound debridement, four out of five observers scored >0.7 for the clinical characteristic of granulation tissue. None of the other items resulted in three or more observers with scores >0.7.

### Image quality

Mean image quality rating on a scale of 1–5, with lower scores reflecting higher quality ratings, was 2.4 (standard deviation 0.3; range 1.8–3.0). Inter-observer reliability kappa on image quality assessment was 0.25, indicating limited agreement between observers on image quality.

## Discussion

We comprehensively investigated the validity and reliability of remote diabetic foot ulcer assessment of clinical characteristics and treatment decisions using mobile phone images for the first time. With the exception of the treatment decision of peri-wound debridement, no other item resulted in strong validity or adequate reliability. This indicates that mobile phone images should not be used as stand-alone diagnostic instrument for remote assessment of diabetic foot ulcers.

This is the first study to investigate both validity and reliability of mobile phone images for remote diabetic foot ulcer assessment. One earlier study investigated clinical assessment of diabetic foot ulcers using mobile phone images^20^, but they reported kappa values for validity rather than the technically correct statistics for validity of likelihood ratio, sensitivity or specificity^21,22^. However, interestingly they also reported overall low values for clinical characteristics using kappa statistics^20^, as we did for clinical characteristics and treatment decisions using likelihood ratio, sensitivity and specificity. The scarce investigations using more advanced wound assessment platform cameras also resulted in low validity for assessment of the same detailed clinical characteristics that we investigated with mobile phone images^14–16^. This confirms that images should not be used as a stand-alone instrument for diagnosis of detailed clinical characteristics or treatment decisions in people with diabetic foot ulcers.

Clinicians who use mobile phone images in daily clinical practice should obtain as much additional information as possible when making treatment decisions based on these images, and be cautious of the low diagnostic accuracy. Evidence-based clinical assessment by a trained health professional commonly includes assessment of presence of peripheral neuropathy, peripheral artery disease, infection, wound size and depth, and, if available, results from radiological or microbiological assessments^25,27^. When combined with such additional clinical information, mobile phone images become part of a more comprehensive telemedicine system.

Two clinical trials have used digital images as part of such a comprehensive telemedicine system to improve treatment decisions for diabetic foot ulcers^31,32^. In one study, a telemedicine system using digital images was added to standard clinical care to help improve treatment decisions for patients in remote Australian clinical sites^31^. In the other study, intervention patients received two home treatments using digital images transferred via a telemedicine system to an expert clinic for advice along with one outpatient clinic treatment, compared to standard care patients receiving three outpatient clinic treatments^32^. Despite the shortcoming in diagnostic accuracy of digital images, these studies reported improved^31^ or similar^32^ outcomes to standard clinical care. However, as the authors highlight, this was likely the result of how they used the images as part of an extensive communication platform between trained nurses and specialised diabetic foot clinicians^31,32^, rather than using the images as the sole diagnostic modality^31,32^. The importance of using an extensive communication platform approach with trained clinicians at both ends of the telemedicine system was further highlighted by a recent telemedicine study that had to be prematurely concluded as clinicians were not confident recruiting patients for the study as it did not have an extensive communication platform approach available^33^. These collective findings indicate that if telemedicine approaches are to be truly effective in diabetic foot ulcer care, and for them to also facilitate improved patient self-care, then telemedicine approaches need images or systems with better diagnostic accuracy and extensive communication platforms between the expert clinic and the remote clinicians or patients that includes additional clinical information.

To improve diagnostic accuracy, other methods are needed to complement the digital images. Some complementary methods have already been described in more advanced systems. For example, infra-red temperature measurement in combination with a digital image holds promise, both to improve diagnosis of infection^18^, as well as to determine urgency of treatment^34^. Small infrared cameras compatible with mobile phones are now on the market, and these have shown adequate quality for diabetic foot ulcer imaging^35^. Another solution to improve the diagnostic accuracy of mobile phone images is to use computerized machine-learning algorithms. Artificial intelligence systems have recently been found to have similar diagnostic accuracy in identifying three types of skin cancers compared to highly trained dermatologists, by making use of computerized machine-learning algorithms^36^. However, the network used 129,450 images to train itself; such a database with reliably annotated diabetic foot ulcers is currently not available. Further, the different clinical characteristics diagnosed that are important for diabetic foot ulcers might present with greater variation between patients and could be harder to detect than the three skin cancer types^36^. With continuously increasing computer power and better availability of diabetic foot ulcer images, this is an area worthy of future research.

Other methods to improve diagnostic accuracy that could be considered are training of assessors and improving image quality and resolution. Studying the effects of additional training may improve diagnostic accuracy, for example the current dataset could be used as a training set, and newly acquired images following training as a test set. However, we did not find any difference in accuracy between experienced and newly-graduated observers, so the effect of training might be limited. All photos in this study were taken with an i-Phone 4. Newer mobile phone models with better cameras may result in images of higher-quality. Assessment of such images might result in better diagnostic accuracy, but this could not be investigated in the current study. With the limited diagnostic accuracy found in studies using more advanced cameras^15,16,20^, it is unlikely that better mobile phone cameras will greatly improve diagnostic accuracy. Also, it cannot be expected that patients will have the newest mobile phone models, and as such the current method was better reflective of daily practice. In our opinion, it would be more relevant to investigate methods that can complement mobile phone images in any future studies, rather than investigating images of higher resolution.

Strengths of our study included involving multiple remote observers with different representative levels of experience in diabetic foot ulcer management. We further mimicked remote assessment similar to daily clinical practice, with inexperienced research assistants taking the photos (reflective of inexperienced patients, carers or home care nurses) and remote observers not involved in the care of the participants included. This is likely the practical daily clinical situation in countries with vast geographical distances and limited specialised interdisciplinary teams, such as Australia, Norway or Canada. Finally, the participants were representative of the target population, with the majority being male, mean age around 61 years, mean average diabetes duration of 20 years, half of the ulcers neuroischemic and 60% located on the plantar side of the foot^8^.

Limitations of our study included the use of live clinical assessment as reference standard. Even though it is the accepted reference standard^27^, live assessment agreement may vary between observers. However, two recent studies (of which one in the same region as the current study), showed adequate to near perfect inter-observer agreement for live assessment^25,37^. The intra-class correlations found in those studies were much higher than what we found for remote assessment of digital images. Another limitation was the lack of information available on ulcer size, depth and duration for the remote observers. It was decided not to include this information from the QHRFF to not bias the observers, and such information would also not always be available in clinical practice with patients taking photos at their home. Future studies might investigate whether availability of such information improves observer agreement. Finally, some variation was seen in image quality in our study, but most observers perceived that the image quality allowed them to adequately assess the ulcer. With the minor differences in quality, a relevant comparison between images with a higher quality and images with a lower quality was not possible.

It is important for these negative outcomes to be reported, as mobile phone images are, in our experience, already widely used in daily clinical practice for the assessment of diabetic foot ulcers and wounds in general. Mobile phone images are often used in addition to verbal descriptions of diabetic foot ulcers when a patient, carer or home care nurse seeks remote assistance from a specialized team. And even though these images may tell more than the words used to describe the ulcer, the low diagnostic values found for both diagnosis of clinical characteristics and for treatment decisions are an important warning that caution is needed when clinicians remotely assess such images.

## Conclusions

With their low validity and reliability, mobile phone images should not be used as a stand-alone diagnostic instrument for remote assessment of diabetic foot ulcers. Clinicians who use mobile phone images in daily clinical practice should obtain as much additional information as possible when making treatment decisions based on these images, and be cautious of the low diagnostic accuracy. Additional methods may improve the diagnostic accuracy, but these need to be developed further before they can be used in daily clinical practice.

## Electronic supplementary material

Supplementary
