# Supplementary material for: The validity and reliability of remote diabetic foot ulcer assessment using mobile phone images
Source: Sci Rep. 2017 Aug 25;7:9480. doi: 10.1038/s41598-017-09828-4 (PMC5573347; doi:10.1038/s41598-017-09828-4)
Supplement: Supplementary file 1 — Supplementary [file 41598_2017_9828_MOESM1_ESM.pdf]

The validity and reliability of remote diabetic foot ulcer assessment using mobile phone images

Jaap J. van Netten[1,2], Damien Clark[2,3], Peter A. Lazzarini[1,2,3,4], Monika Janda[5], Lloyd F. Reed[1]

1 – School of Clinical Sciences, Queensland University of Technology, Brisbane, QLD, Australia

2 – Wound Management Innovation Cooperative Research Centre, West End, QLD 4101, Australia

3 – Department of Podiatry, Metro North Hospital & Health Service, Queensland Health, Brisbane, QLD, Australia

4 – Allied Health Research Collaborative, Metro North Hospital & Health Service, Queensland Health, Brisbane, QLD, Australia

5 - School of Public Health and Social Work, Queensland University of Technology, Brisbane, QLD, Australia

Supplementary information

Supplementary Table 1: Prevalence of clinical characteristics and treatment decisions during live and remote assessment

**Clinical characteristics** [a]	Live		Obs.1		Obs.2		Obs.3		Obs.4		Obs.5	
Granulation	66	(33)	66	(33)	66	(33)	82	(41)	48	(24)	68	(34)
Ischemia	22	(11)	54	(27)	86	(43)	72	(36)	36	(18)	16	(8)
Infection	18	(9)	48	(24)	30	(15)	68	(34)	18	(9)	24	(12)
Slough	42	(21)	64	(32)	14	(7)	58	(29)	50	(25)	60	(30)
Tendon or bone	0	(0)	0	(0)	8	(4)	2	(1)	0	(0)	0	(0)
Tracking or tunnelling	28	(14)	22	(11)	24	(12)	32	(16)	4	(2)	18	(9)
Moist or exuding	44	(22)	56	(28)	74	(37)	68	(34)	58	(29)	80	(40)
Wet or dry gangrene	0	(0)	2	(1)	2	(1)	14	(7)	0	(0)	4	(2)
Cellulitis or erythema	22	(11)	46	(23)	6	(3)	30	(15)	18	(9)	12	(6)
**Treatment decisions** [a]												
Wound debridement	70	(35)	92	(46)	62	(31)	88	(44)	98	(49)	68	(34)
Peri-wound debridement	70	(35)	100	(50)	82	(41)	98	(49)	96	(48)	78	(39)
Urgent treatment	44	(22)	0	(0)	20	(10)	66	(33)	34	(17)	22	(11)

Note: Values are % (n). [a]: See table 1 for formulation of the questions, all answers were 'yes' or 'no' with percentage 'yes' given in this table; there were no missing observations, so all total to n=50. [b]: First remote assessment.

Supplementary Table 2: Validity of remote assessment of clinical characteristics and treatment decisions

**Absolute agreement**	**Obs.1**	**Obs.2**	**Obs.3**	**Obs.4**	**Obs.5**
**Clinical characteristics** [a]					
Granulation	84	84	68	54	86
Ischemia	64	32	46	62	78
Infection	66	84	50	68	78
Slough	70	64	80	60	66
Tracking or tunnelling	74	72	68	68	82
Moist or exuding	80	66	68	42	60
Cellulitis or erythema	64	76	72	60	78
Mean per observer	*71.7*	*68.3*	*64.6*	*59.1*	*75.4*
**Treatment decisions** [a]					
Wound debridement	74	56	74	68	70
Peri-wound debridement	70	76	68	74	88
Urgent treatment	56	68	62	38	58
Mean per observer	*66.7*	*66.7*	*68.0*	*60.0*	*72.0*
**Sensitivity**	**Obs.1**	**Obs.2**	**Obs.3**	**Obs.4**	**Obs.5**
**Clinical characteristics** [a]					
Granulation	88 [72;97]	88 [72;97]	88 [72;97]	52 [34;69]	91 [76;98]
Ischemia	91 [59;100]	91 [59;100]	91 [59;100]	45 [17;77]	36 [11;69]
Infection	89 [52;100]	89 [52;100]	100 [66;100]	11 [0;48]	56 [21;86]
Slough	90 [70;99]	24 [8;47]	95 [76;100]	62 [38;82]	81 [58;95]
Tracking or tunnelling	43 [18;71]	43 [18;71]	50 [23;77]	0 [0;23]	50 [23;77]
Moist or exuding	91 [71;99]	95 [77;100]	91 [71;99]	50 [28;72]	95 [77;100]
Cellulitis or erythema	73 [39;94]	9 [0;41]	55 [23;83]	0 [0;28]	27 [6;61]
Mean per observer	*80.7*	*62.7*	*81.4*	*31.4*	*62.3*
**Treatment decisions** [a]					
Wound debridement	97 [85;100]	63 [45;79]	94 [81;99]	97 [85;100]	77 [60;90]
Peri-wound debridement	100 [90;100]	91 [77;98]	97 [85;100]	100 [90;100]	97 [85;100]
Urgent treatment	0 [0;15]	36 [17;59]	82 [60;95]	18 [5;40]	27 [11;50]
Mean per observer	*65.7*	*63.3*	*91.0*	*71.7*	*67.0*
**Specificity**	**Obs.1**	**Obs.2**	**Obs.3**	**Obs.4**	**Obs.5**
**Clinical characteristics** [a]					
Granulation	76 [50;93]	76 [50;93]	29 [10;56]	59 [33;82]	76 [50;93]
Ischemia	56 [40;72]	15 [6;31]	33 [19;50]	67 [50;81]	90 [76;97]
Infection	61 [45;76]	83 [68;93]	39 [24;55]	80 [65;91]	83 [68;93]
Slough	55 [36;74]	93 [77;99]	69 [49;85]	59 [39;76]	55 [36;74]
Tracking or tunnelling	86 [71;95]	83 [67;94]	75 [58;88]	94 [81;99]	94 [81;99]
Moist or exuding	71 [51;87]	43 [24;63]	50 [31;69]	36 [19;56]	32 [16;52]
Cellulitis or erythema	62 [45;77]	95 [83;99]	77 [61;89]	77 [61;89]	92 [79;98]
Mean per observer	*66.7*	*69.7*	*53.1*	*67.4*	*74.6*
**Treatment decisions** [a]					
Wound debridement	20 [4;48]	40 [16;68]	27 [8;55]	0 [0;22]	53 [27;79]
Peri-wound debridement	0 [0;22]	40 [16;68]	0 [0;22]	13 [2;40]	67 [38;88]
Urgent treatment	100 [88;100]	93 [76;99]	46 [28;66]	54 [34;72]	82 [63;94]
Mean per observer	*40.0*	*57.7*	*24.3*	*22.3*	*67.3*

Note: values are % [95% confidence interval]. [a]: See table 1 for formulation of the questions, all answers were ‘yes’ or ‘no’ with percentage ‘yes’ given in this table.

Supplementary Table 3: Test-retest reliability per observer

**Clinical characteristics** [a]	Obs.1		Obs.2		Obs.3		Obs.4		Obs.5	
	S	%	S	%	S	%	S	%	S	%
Granulation	0.80	90	0.92	96	0.84	92	-0.24	38	0.80	90
Ischemia	0.52	76	0.76	88	0.52	76	0.36	68	0.20	60
Infection	0.48	74	0.64	82	0.39 [b]	69 [b]	0.24	62	0.60	80
Slough	0.36	68	0.88	94	0.31 [b]	65 [b]	0.16	58	0.56	78
Tracking or tunnelling	0.48	74	0.56	78	0.60	80	0.76	88	0.80	90
Moist or exuding	0.48	74	0.72	86	0.64	82	0.00	50	0.64	82
Cellulitis or erythema	0.44	72	0.88	94	0.52	76	0.28	64	0.56	78
**Treatment decisions** [a]										
Wound debridement	0.76	88	0.44	72	0.68	84	0.96	98	0.60	80
Peri-wound debridement	1.00	100	0.72	86	0.92	96	0.88	94	0.76	88
Urgent treatment	1.00	100	0.52	76	0.60	80	-0.24	38	0.52	76

Note: S = free marginal Randolph's kappa coefficient. Obs. = Observer. % = percentage agreement. [a]: See table 1 for formulation of the questions, all answers were 'yes' or 'no' with percentage 'yes' given in this table.[b]: second observation missing, so n=49.
